# Preliminary Post-Dobbs Trends in Emergency Department Use for Early Pregnancy Complications

**DOI:** 10.5811/westjem.50661

**Published:** 2026-01-09

**Authors:** Emily E. Ager, Ralph Wang, Lyndsey S. Benson

**Affiliations:** * University of California San Francisco School of Medicine, Department of Emergency Medicine, San Francisco, California; † University of Washington School of Medicine, Department of Obstetrics and Gynecology, Seattle, Washington

## Abstract

**Introduction:**

Following the 2022 Dobbs Supreme Court decision, emergency department (ED) use for early pregnancy complications (EPC), such as miscarriage and ectopic pregnancy, may increase in states that enacted severely restrictive abortion policies. Patients may increasingly seek EPC-related care in the ED due to delays in treatment resulting in greater symptom severity or reduced access to usual settings of obstetric and family planning care. Our objective in this study was to examine the association between severely restrictive abortion policies and post-Dobbs EPC-related ED visits.

**Methods:**

This retrospective, cross-sectional study used data from the 2016–2022 National Hospital Ambulatory Medical Care Survey. Our primary outcome measure was ED visits among female patients 15–49 years of age for EPC-related care, defined using encounter diagnosis code or chief complaint. We used multivariable logistic regression to examine the association between US region and post-Dobbs, EPC-related visits, as the number of states with severely restrictive abortion policies (bans from conception to six weeks) varied by region, from zero (Northeast) to 10 of 17 states in the South.

**Results:**

We identified 7,872,445 weighted EPC-related visits (unweighted n = 1,008) among 266,222,232 weighted (unweighted n = 32,841) encounters for female patients 15–49 years of age (3.0%). The median age was 28 (IQR 23–32). The proportion of pre- vs post-Dobbs EPC-related visits was 3.1% vs 2.5% in the Northeast (P = .72); 3.2% vs 3.5% in the West (P = .80); 2.4% vs 3.1% in the Midwest (P = .36); and 2.9% vs 3.7% in the South (P = .50). Compared to the Northeast, the adjusted odds of post-Dobbs EPC-related visits were 1.4 in the West (95% CI, 0.4–5.2), 1.4 in the Midwest (95% CI, 0.4–4.6), and 1.3 in the South (95% CI, 0.4–4.7).

**Conclusion:**

This preliminary study did not find a statistically significant association between US region and post-Dobbs ED visits for early pregnancy complications. However, given the increasing restrictions surrounding reproductive healthcare access, the ED represents an important setting for the delivery of this care. Further investigations using more robust data sources are needed to understand the effect of prohibitive abortion laws on the ED use and the management of early obstetric complications.

## INTRODUCTION

The 2022 US Supreme Court decision in Dobbs v Jackson Women’s Health Organization allowed individual states to regulate abortion access.^1^ Following this decision, many states immediately enacted severely restrictive abortion policies. These states were disproportionately in the South and Midwest.^2^ Prohibitive abortion policies can impact treatment for early pregnancy complications (EPC), such as ectopic pregnancy or miscarriage. Clinicians may fear legal consequences for providing treatment involving pregnancy termination, potentially leading to undertreatment or treatment delays.^3^–^5^ Further, eliminating family planning care settings can decrease access to prenatal and gynecological services.^3^,^6^

As a result of these changes, individuals in abortion prohibitive states many increasingly seek EPC-related care in the emergency department (ED).^7^ Patients not offered treatment for miscarriage or ectopic pregnancy may develop worsening symptoms necessitating emergent evaluation.^7^ Definitive treatment may be delayed until patients meet state exceptions for abortion, increasing the risk of complications requiring ED management.^5^ Decreased access to usual settings of obstetric care may lead patients to seek ED care for early pregnancy concerns.^7^–^9^ Self-managed abortion has increased post-Dobbs, and ED visits for complications, although rare, may increase.^10^

The impact of severely restrictive abortion policies on ED utilization for EPC-related care is currently unknown. In this study we aimed to examine the association between severe abortion restrictions and post-Dobbs EPC-related ED encounters.

## METHODS

### Study Design and Data Source

This was a retrospective, cross-sectional study of patient encounters at US hospital-based EDs from 2016–2022. We used data from the National Hospital Ambulatory Medical Care Survey (NHAMCS), a nationally representative sample of ED visits collected annually by the National Center for Health Statistics at the Centers for Disease Control and Prevention. The NHAMCS uses a four-stage probability sampling design, and sampling weights are provided to produce population-based estimates. Non-federal, general, and acute care hospitals located in the 50 US states and the District of Columbia are eligible for sampling. We chose 2016 as the start of the study period given the transition to the *International Classification of Diseases, 10th Revision*. (*ICD-10*), in 2016. The final year of NHAMCS collection was 2022. This study adheres to STROBE guidelines for observational studies (Appendix 1).^11^ In this study we used the publicly available, de-identified NHAMCS; our study was exempt from review by the University of California, San Francisco Institutional Review Board.

### Study Population

We identified all ED visits in NHAMCS during the study period of 2016–2022. We then restricted the study population to patients coded as female and those of reproductive age, defined as 15–49 years of age, Because of the limitations of NHAMCS, we could not capture ED encounters among pregnant patients coded as other than female.

### Outcome Measures

The primary outcome was the proportion of ED visits for EPC-related care among all visits for female patients of reproductive age. We identified EPC-related visits using relevant *ICD-10* and reason for visit codes (Appendix 2).^8^,^12^ These codes encompass encounters for bleeding in pregnancy, the spectrum of miscarriage and abortion, ectopic and molar pregnancy, and other abnormal products of conception. We described patient demographics characteristics for these visits. These include patient age, race, ethnicity, and insurance payer. Age was categorized as ≥ 35 years vs <35 years, as advanced maternal age (≥ 35 years) is a risk factor for miscarriage and ectopic pregnancy. We describe EPC-related encounter characteristics, including hospitalization, repeat ED visit within 72 hours, and Metropolitan Statistical Area status.

**Population Health Research Capsule**

What do we already know about this issue?

*Following the enactment of restrictive abortion policies, predominantly in the South, delays in management for early pregnancy loss and ectopic pregnancy were reported*.

What was the research question?

*Are severely restrictive abortion policies associated with an increase in emergency department (ED) encounters for early obstetric complications?*

What was the major finding of the study?

*Post-Dobbs, there was a non-significant rise in ED visits for early obstetric complications in the South (2.9% before vs 3.7% after; P = .50)*.

How does this improve population health?

*This preliminary study provides initial evidence on how severe abortion restrictions may lead to changes in ED use for early pregnancy complications; further studies are needed*.

### Exposure

We categorized the study period as before (January 2016–June 2021) or after (July–December 2022) the Dobbs decision. The public NHAMCS does not include state-level data. We thus used US region as a proxy for exposure to severely restrictive abortion policies, defined as bans from conception to six weeks, as varying numbers of states in each region enacted such policies immediately following the Dobbs decision (Appendix 3). This included zero of nine Northeastern states, one of 13 (8%) Western states, three of 12 (25%) Midwestern states, and 10 of 17 (59%) Southern states (Appendix 3).

### Analysis

We performed a descriptive analysis of EPC-related ED visits, including the proportion of these visits among all visits for females ages 15–49 years of age, patient demographics, and encounter characteristics. Next, we performed a bivariate analysis comparing the proportion of EPC-related visits before (July-December 2016–2021) and after (July-December 2022) the Dobbs decision, overall and by US region. We restricted the pre-Dobbs period to July-December to account for seasonality in ED visits. We used survey-weighted Pearson chi-squared statistics, and we report associated *P*-values. Statistical significance was set at *P* < .05. The NHAMCS multistage probability-based sample weights were applied to obtain unbiased population-level estimates. Weighted results are reported unless otherwise specified.

We then used multivariable logistic regression to examine the association between US region and post-Dobbs, EPC-related visits. Our model included an interaction term between time-period (pre- [January 2016–June 2022] vs post- [July-December 2022] Dobbs) and region; Northeast is the reference as no states enacted a total or six-week abortion ban. We examined unadjusted associations, and then estimated multivariable logistic regression models adjusting for age, race, ethnicity, insurance payor, and season.^12^ We conducted a sensitivity analysis excluding 2020–2021 given ED use variability during the Covid-19 pandemic. Total case analysis was performed. We report odds ratios (OR) and 95% confidence intervals. Analyses were performed using Stata 18.5 (StataCorp, College Station, TX).

## RESULTS

During the study period, there were 7,872,445 weighted EPC-related visits (unweighted n = 1,008) among 266,222,232 weighted visits (unweighted n = 32,841) for female patients 15–49 years of age (3.0%). By region, EPC-related visits accounted for 1,268,956 weighted encounters in the Northeast (3.1%), 1,749,370 encounters in the West (3.0%), 1,517,557 encounters in the Midwest (2.6%), and 3,336,563 encounters in the South (3.1%). Patient and encounter characteristics, overall and by US region, are displayed in Table 1.

The overall proportion of EPC-related visits in the pre-Dobbs period (restricted to July-December) was 2.9% (3,271,128 weighted visits) and 3.4% (752,526 visits) in the post-Dobbs period (*P* = .44). By US region, the proportion of pre- vs post-Dobbs EPC-related encounters was 3.1% (559,291 visits) vs 2.5% (58,667 visits) in the Northeast (*P* = .72); 3.2% (757,876 visits) vs 3.5% (130,945 visits) in the West (*P* = .80); 2.4% (555,856 visits) vs 3.1% (153,327 visits) in the Midwest (*P* = .36); and 2.9% (1,398,105 visits) vs 3.7% (409,587 visits) in the South (*P* = .50) (Appendix 4).

In the fully adjusted logistic regression model, compared to the Northeast region, the odds of post-Dobbs EPC-related visits were 1.37 times greater in the West (95% CI, 0.36–5.20), 1.39 times greater in the Midwest (95% CI, 0.42–4.62), and 1.34 times greater in the South (95% CI, 0.39–4.65) (Table 2). A sensitivity analysis excluding 2020–2021 did not lead to significantly different point estimates.

## DISCUSSION

This is the first study to examine national trends in ED utilization following the Dobbs decision. In this preliminary analysis, we did not find clear evidence for increased EPC-related ED visits by US region in the six months following the Dobbs decision. Estimates from our multivariable regression analysis demonstrate an increased odds of EPC-related visits in regions with a greater number of states with total or six-week abortion bans. However, the 95% CIs for these estimates are too wide to rule out the possibility of no effect.

The proportion of EPC-related encounters increased in the post-Dobbs period in all regions except the Northeast, although these differences did not reach statistical significance. The greatest increases were in the South (2.9% to 3.7%; *P* = .50) and Midwest (2.4% to 3.1%; *P* = .36). Emergency department visits related to EPC continue to represent approximately 3% of all visits among females of reproductive age. This finding is in line with previous estimates, representing over one million annual visits.^12^,^13^

While literature on this topic is nascent, a recent national survey of emergency physicians found that 20% of participants in abortion-permissive states reported increased pregnancy-related visits among patients from abortion-restrictive states.^14^ Among participants in abortion-restrictive states, 24% reported delays in their management of suspected or known ectopic pregnancy.^14^ Such delays could result in repeat ED utilization for ongoing symptoms. After Texas enacted a total abortion ban in 2022, miscarriage-related ED visits rose by 25%, and blood transfusions during such visits increased by 54%, further supporting the occurrence of delays in care in abortion-restrictive settings leading to ED visits for life-threatening complications.^15^

## LIMITATIONS

This study has several limitations. First, we used survey-based data from NHAMCS, which has several known limitations.^16^ Next, this analysis lacked long-term post-Dobbs data, as 2022 was the final year NHAMCS was collected. As there were only 53 unweighted post-Dobbs EPC-related visits, this analysis was underpowered to detect a significant change in our primary outcome, resulting in wide confidence intervals in our regression analysis. Finally, the public-use NHAMCS does not include state-level data. Region was instead used as the exposure, as severely restrictive policies were disproportionately enacted in the South and Midwest. Data sources with further post-Dobbs data and state-level estimates may yield a more definitive comparison.

## CONCLUSION

This preliminary study did not find evidence for changes in early pregnancy-related ED utilization by region immediately following the Dobbs decision, although the study is limited in several ways. Nevertheless, the ED represents an increasingly important setting for the delivery of reproductive health care. Further studies using robust data sources are needed to investigate trends in pregnancy-related visits in abortion restrictive states, including the management and outcomes for these conditions.

## Supplementary Information









## Figures and Tables

**Table 1:** Characteristics of emergency department encounters for early pregnancy complications among female patients 15–49 years of age, by US region (2016–2022), weighted.

	All N = 7,872,445	Northeast n = 1,268,956 (15.2%)	West n = 1,749,370 (21.9%)	Midwest n = 1,517,557 (21.9%)	South n = 3,336,563 (41.0%)
Age (median [IQR])	28 (23–32)	29 (24–37)	29 (25–33)	23 (27–31)	27 (22–31)
Age (years)					
Age < 35	6,407,229 (81.39)	887,155 (69.91)	1,371,291 (78.39)	1,309,173 (86.27)	2,839,610 (85.11)
Age ≥ 35	1,465,216 (18.61)	381,801 (30.09)	378,079 (21.61)	208,384 (13.73)	496,952 (14.89)
Race and Ethnicity					
Non-Hispanic White	3,471,488 (44.10)	476,895 (37.58)	742,967 (42.47)	476,895 (49.72)	1,497,133 (44.87)
Non-Hispanic Black	2,147,249 (27.28)	399,688 (31.50)	71,808 (4.10)	399,688 (31.66)	1,195,307 (35.82)
Hispanic	1,916,896 (24.35)	350,483 (27.62)	816,358 (46.67)	350,483 (14.73)	526,581 (15.78)
Other	336,811 (4.28)	41,890 (3.30)	118,237 (6.76)	41,890 (3.90)	117,542 (3.52)
Insurance payor					
Self-pay	786,121 (10.92)	68,289 (6.07)	109,015 (6.71)	120,887 (9.33)	487,931 (15.46)
Private insurance	2,168,211 (30.11)	440,739 (39.20)	512,119 (31.52)	427,884 (33.03)	787,470 (24.94)
Medicaid or Medicare	4,030,909 (55.98)	587,409 (52.24)	949,123 (58.43)	738,831 (57.04)	1,755,546 (55.61)
Other/unknown	215,914 (3.00)	27,954 (2.49)	54,250 (3.34)	7,676 (0.59)	126,035 (3.99)
MSA status					
MSA	7,144,726 (90.76)	1,234,303 (97.27)	1,710,765 (97.79)	1,310,087 (86.33)	2,889,571 (86.60)
Non-MSA	727,719 (9.24)	34,653 (2.73)	38,605 (2.21)	207,470 (13.67)	446,991 (13.40)
Hospital admittance					
Yes	407,748 (5.18)	78,474 (6.18)	112,432 (6.43)	73,555 (4.85)	143,287 (4.29)
No	7,464,696 (94.82)	1,190,482 (93.82)	1,636,938 (93.57)	1,444,002 (95.15)	3,193,275 (95.71)
72-hour revisit					
Yes	435,049 (5.53)	69,050 (5.44)	122,538 (7.00)	38,128 (2.51)	205,334 (6.15)
No	6,768,088 (85.97)	1,136,568 (89.57)	1,423,230 (81.36)	1,436,239 (94.64)	2,772,051 (83.08)
Unknown/blank	669,308 (8.50)	63,338 (4.99)	203,602 (11.64)	43,190 (2.85)	359,178 (10.76)

Note: Numbers may not sum to 100% due to rounding error.

*MSA*, Metropolitan Statistical Area.

**Table 2:** Unadjusted and multivariable logistic regression results: association between United States region and post-Dobbs period on EPC-related ED visits related to early pregnancy complications among female patients 15–49 years of age (2016–2022).

	OR	95% CI	aOR^a^	95% CI	aORb	95% CI
Region*/Time Period						
Northeast	REF	-	REF	-	REF	-
West	1.53	(0.39, 0.93)	1.49	(0.38, 5.79)	1.37	(0.36, 5.20)
Midwest	1.52	(0.44, 5.21)	1.50	(0.44, 5.10)	1.39	(0.42, 4.62)
South	1.57	(0.44, 5.63	1.53	(0.44, 5.34)	1.34	(0.39, 4.65)
Time Period						
Pre-Dobbs	REF	-	REF	-	REF	-
Post-Dobbs	0.79	(0.28, 2.25)	0.80	(0.28, 2.30)	0.84	(0.30, 2.35)
Region						
Northeast	REF	-	REF	-	REF	-
West	0.93	(0.68, 1.28)	0.94	(0.68, 1.28)	0.84	(0.61, 1.15)
Midwest	0.80	(0.60, 1.07)	0.81	(0.61, 1.07)	0.82	(0.61, 1.11)
South	0.94	(0.71, 1.25)	0.94	(0.71, 1.25)	0.98	(0.74,1.30)

a Adjusted for season (Summer: June, July, August; Fall: September, October, November; Winter: November, December, January; Spring: March, April, May).

b Adjusted for season, age, race and ethnicity, and payment.

*EPC*, early pregnancy complications; *OR*, odds ratio; *REF*, reference.
