# Supplementary material for: Preliminary Post-Dobbs Trends in Emergency Department Use for Early Pregnancy Complications
Source: West J Emerg Med. 2026 Jan 9;27(1):85–90. doi: 10.5811/westjem.50661 (PMC12815543; doi:10.5811/westjem.50661)
Supplement: Supplementary file 2 [file wjem-27-85-s002.docx]

**Appendix 2.** International Classification of Diseases, Tenth Revision, Clinical Modification (ICD-10-CM) diagnosis codes and Reason for visit (RFV) codes consistent with care related to early pregnancy complications.

| **Definition** | **Code Type** | **Code** |
| --- | --- | --- |
| Ectopic pregnancy | ICD-10 | O00.X |
| Hydatidiform mole | ICD-10 | O01.X |
| Other abnormal products of conception | ICD-10 | O02.X |
| Blighted ovum and nonhydatidiform mole | ICD-10 | O02.0 |
| Missed abortion | ICD-10 | O02.1 |
| Other specified abnormal products of conception | ICD-10 | O02.8 |
| Abnormal product of conception, unspecified | ICD-10 | O02.9 |
| Spontaneous abortion | ICD-10 | O03.X |
| Complications following (induced) termination of pregnancy | ICD-10 | O04.X |
| Failed attempted termination of pregnancy | ICD-10 | O07.X |
| Complications following ectopic and molar pregnancy | ICD-10 | O08.X |
| Hemorrhage in early pregnancy | ICD-10 | O20.X |
| Threatened abortion | ICD-10 | O20.0 |
| Other hemorrhage in early pregnancy | ICD-10 | O20.8 |
| Hemorrhage in early pregnancy, unspecified | ICD-10 | O20.9 |
| Spotting or bleeding during pregnancy | RFV | 17902 |

Abbreviations: ICD, international classification of disease, RFV, reason for visit.
