# Supplementary material for: Preliminary Post-Dobbs Trends in Emergency Department Use for Early Pregnancy Complications
Source: West J Emerg Med. 2026 Jan 9;27(1):85–90. doi: 10.5811/westjem.50661 (PMC12815543; doi:10.5811/westjem.50661)
Supplement: Supplementary file 3 [file wjem-27-85-s003.docx]

**Appendix 3.** U.S. states completely prohibiting abortion or banning abortion to less than 6 weeks gestational duration immediately following the June 2022 Dobbs v. Jackson Women's Health Organization Supreme Court Decision, by U.S. region.

| **U.S. Region** | **U.S. State** |
| --- | --- |
| Northeast | N/A |
| West | Idaho |
| Midwest | Missouri |
|  | South Dakota |
|  | Wisconsin |
| South | Alabama |
|  | Arkansas |
|  | Georgia |
|  | Kentucky |
|  | Louisiana |
|  | Mississippi |
|  | Oklahoma |
|  | Tennessee |
|  | Texas |
|  | West Virginia |
