# Supplementary material for: Preliminary Post-Dobbs Trends in Emergency Department Use for Early Pregnancy Complications
Source: West J Emerg Med. 2026 Jan 9;27(1):85–90. doi: 10.5811/westjem.50661 (PMC12815543; doi:10.5811/westjem.50661)
Supplement: Supplementary file 4 [file wjem-27-85-s004.docx]

**Appendix 4.** Proportion EPC-related ED visits among female patients ages 15-49, by pre- (Jul-Dec 2017 - 2021) vs. post- (Jul-Dec 2022) Dobbs period, weighted.

|  | **Pre-Dobbs**  **No. (%)** | **Post-Dobbs**  **No. (%)** | **p-value^a^** |
| --- | --- | --- | --- |
| All regions | 3,271,128  (2.87) | 752,526  (3.39) | .44 |
| Northeast | 559,291  (3.05) | 58,667  (2.51) | .72 |
| West | 757,876  (3.18) | 130,945  (3.54) | .80 |
| Midwest | 555,856  (2.36) | 153,327  (3.06) | .36 |
| South | 1,398,105  (2.90) | 409,587  (3.67) | .50 |

^a^P-values obtained from Pearson’s chi-squared test
